# Canopy‐Mediated Dynamics of Moss Communities in Primary Succession: Coupling of N_2_ ‐Fixation and Biomass Accumulation in Subalpine Forests Following Glacial Retreat

**DOI:** 10.1002/ece3.71763

**Published:** 2025-07-11

**Authors:** Jie Deng, Genxu Wang, Shouqin Sun, Wentian Xie, Feng Long, Zhaoyong Hu, Juying Sun, Xiangyang Sun, Thomas H. DeLuca

**Affiliations:** ^1^ State Key Laboratory of Hydraulics and Mountain River Engineering, College of Water Resource and Hydropower Sichuan University Chengdu China; ^2^ College of Forestry Oregon State University Corvallis Oregon USA

**Keywords:** bryophyte, canopy‐bottom layer interactions, glacial primary succession, leaf litter, N_2_‐fixaiton, photosynthesis

## Abstract

Accelerated glacial retreat has exposed bare substrates in polar and alpine regions, creating opportunities for investigating primary vegetation succession. Mosses are pioneer species critical for soil development, nutrient cycling, and establishment of subsequent vegetation succession. However, the dynamics of moss communities during primary succession and their responses to canopy‐mediated environmental changes are poorly known. We investigated moss bottom community dynamics along a 129‐year primary successional gradient from barren land to coniferous climax forest on a deglacial foreland in eastern Qinghai‐Tibet Plateau. Additionally, we conducted a reciprocal transplant experiment and a canopy tree litter addition experiment at three succession stages with distinct canopy densities to explore the effects of shifts in canopy composition on the development of the moss bottom layer. Moss biomass and cover in the bottom layer had a nonlinear and fluctuating growth pattern across the primary successional chronosequence, in which successional stages with higher canopy density had lower moss cover and biomass. Transplantation of moss carpets from open to denser canopy stages or canopy litter additions enhanced photosynthetic rates, but suppressed N_2_‐fixation rates and moss growth. Variations in N_2_‐fixation and photosynthesis rates were related to daylight hours, relative humidity, and throughfall N levels. Changes in moss bottom layer cover and biomass over the successional chronosequence were positively related to N_2_‐fixation and regulated by canopy leaf litter and throughfall N inputs. Our results demonstrate a strong coupling between moss biomass and cyanobacterial N_2_‐fixation, alongside a decoupling of moss photosynthesis from productivity during primary succession following glacial retreat. The effects of canopy cover and composition on moss productivity, photosynthesis, and N_2_‐fixation rates represent a dynamic set of canopy‐bottom layer interactions that may shape the structure and function of developing subalpine forest.

## Graphical abstract

This study examined the dynamics of moss communities along a 129‐year primary successional gradient on the eastern Qinghai‐Tibet Plateau and explored the effects of canopy composition changes through transplantation and litter addition experiments. Results showed that increasing canopy density led to decreased moss cover and biomass, while canopy litter and nitrogen inputs influenced moss nitrogen fixation and photosynthesis. The findings highlight a strong coupling between moss biomass accumulation and nitrogen fixation, as well as the complex role of canopy structure in regulating moss productivity and ecological functions.

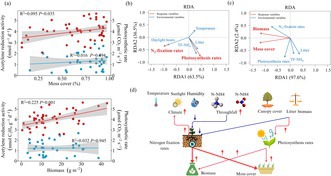

## 1 Introduction

Climate change has accelerated glacial retreat at high latitudes and elevations, exposing terrain devoid of vegetation (Barnett et al. 2005; Barry 2006). These newly revealed moraines in polar and high‐altitude regions have minimal pedogenic development, and thus provide opportunities to study the primary vegetation succession (Crocker and Major 1955; Jones and Henry 2003; Cazzolla Gatti et al. 2018).

Following glacial retreat, mineral substrates are colonized by vascular plants, mosses, lichens, and soil biota within a few years (Burga et al. 2010), forming a complete, age‐determinable chronosequence. Mosses play a crucial role in ecosystem development as they are the earliest colonizers and enhance nutrient accumulation through N_2_‐fixation (Tallis 1958; Klarenberg et al. 2023). They also regulate the microclimate, retain moisture, protect the soil layer, and facilitate the establishment of vascular plants, especially in cold ecosystems (Beringer et al. 2001; Cornelissen et al. 2007). Mosses have high species diversity, higher cover, and a more complex community structure and function during succession, due to their unique traits that promote nutrient buildup and microbial expansion (Turetsky et al. 2010; Laine et al. 2021; Matthews and Vater 2015; Rzepczynska et al. 2022). Previous studies have shown that shifts in canopy composition and cover are followed by shifts in understory species diversity, cover, and community structure (Turetsky et al. 2010; Laine et al. 2021). Yet, little is known about how the canopy and moss bottom layer interact, particularly how shifts in canopy vegetation affect the growth and community dynamics of the moss bottom layer in primary succession.

Mosses have unique adaptations to nutrient‐limited environments (Aldous 2002). Under natural conditions, moss growth and photosynthesis can be supported by atmospheric N deposition. Mosses may also meet their N demands through moss‐associated cyanobacteria N_2_‐fixation (Deluca et al. 2002; Rousk et al. 2013). This N_2_‐fixing action of associated diazotrophic bacteria may enhance the moss growth rate, leading to a strong correlation between moss growth and the amount of fixed N_2_ (Berg et al. 2013). Photosynthesis and N_2_‐fixation rates of moss‐cyanobacteria associations are also sensitive to environmental variables, such as moisture (Gundale et al. 2012; Rousk et al. 2014), temperature (Lindo and Griffith 2017), light availability (Gundale et al. 2012; Permin et al. 2021), and N deposition (Van Gaalen et al. 2007; Cui et al. 2009; Laine et al. 2011; Gundale et al. 2013; Du et al. 2014). For instance, suboptimal temperature and light attenuation due to canopy shifting can suppress moss photosynthesis (Murray et al. 1993; Bjerke et al. 2011, 2013), while increased litter accumulation may alter moisture availability and nutrient fluxes (Li et al. 2017; Wu et al. 2021). Paradoxically, denser canopies may mitigate drought stress in arid regions by prolonging photosynthetic activity (Li et al. 2017), yet simultaneously reduce light penetration and N_2_‐fixation (Gundale et al. 2012; Kox et al. 2020).

Moreover, mosses lack roots and depend on atmospheric and canopy throughfall deposition for nutrients. Elevated N leaching from canopy throughfall and tree litter in denser canopy forests may enhance moss photosynthesis and growth, but downregulate N_2_‐fixation (Gundale et al. 2012; Salemaa et al. 2019). Moreover, phenol‐rich leachates from increased plant litter and physical shading may have adverse effects on moss N_2_‐fixation (Natalia et al. 2008; Sorensen and Michelsen 2011; Sorensen et al. 2012). Conversely, increased phosphorus and labile carbon from litter decomposition could stimulate N_2_‐fixation (Vitousek et al. 2002; Gundale et al. 2009; Lett and Michelsen 2014). Nonetheless, no study has examined how successional shifts in canopy vegetation influence moss physiology and community assembly.

Here, we tested how cover, biomass, photosynthesis, and N_2_ fixation rates vary with moss bottom layer along a primary succession chronosequence on the eastern Tibetan Plateau. Specifically, we conducted a reciprocal transplant experiment and a canopy tree litter addition experiment at key successional stages. Our goals were: (1) to determine the growth dynamics (cover and biomass) of mosses over time since glacial retreat; (2) to evaluate how canopy‐driven microenvironment modification and tree litter inputs affect moss photosynthesis and N_2_ fixation; and (3) to assess how changes in moss cover and biomass correlate with shifts in photosynthetic efficiency and N_2_‐fixation rates during succession.

## 2 Material and Methods

### 2.1 Study Site

**FIGURE 1:**
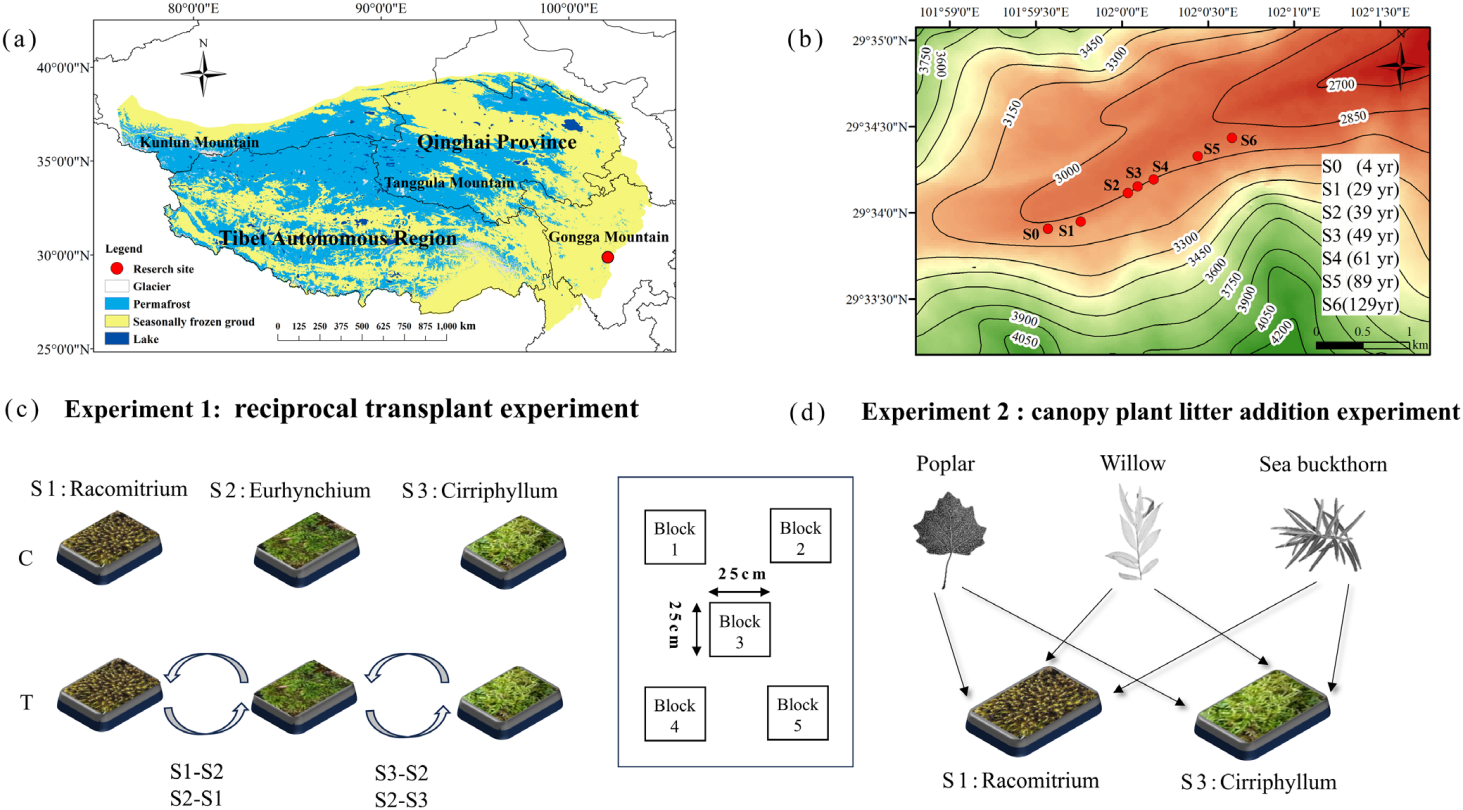
Maps and schematic diagram of study sites on the primary successional chronosequence on the glacial retreat area of Hailuogou. (a) Map of the location of the study sites; (b) Sampling sites (and stand age) on the successional sequence of the glacial retreating area of Hailuogou; (c) Schematic diagram of the experimental design of reciprocal moss‐transplantation; (d) Schematic diagram of the experimental design of the canopy tree litter addition experiment. S1–S2, transplanting moss microcosms from site 1 (S1) to site 2 (S2); S2–S1, from site 2 to site 1; S2–S3, from site 2 to site 3; S3–S2, from site 3 to site 2. B1–B5 represent the five blocks for the reciprocal transplantation and litter addition experiment.

The study was conducted on the foreland of Hailuogou Glacier (29°20′–30°20′N, 101°30′–102°15′E) on the eastern edge of the Qinghai‐Tibet Plateau, China (Figure 1a). The region experiences a typical monsoon temperate climate, with a mean annual temperature of 4.8°C, January (−4.30°C) being the coldest month and July or August (12.97°C) being the hottest ones. The mean annual precipitation is approximately 1861 mm, usually concentrated from June to October, while the annual average relative humidity is 90%. Soils are classified as Regosols according to the World Reference Base for Soil Resources, with a pH of 6.41 ± 1.07.

After 129 years of primary succession, the vegetation has developed into distinct communities ranging from pioneer to climax coniferous communities at seven successional ages (4, 29, 39, 49, 61, 89, and 129 years since the year of glacier retreating to 2019, respectively). We defined these sites S0–S6. The S0 sites (4 years) were almost bare ground, sparsely covered by *Hippophae rhamnoides* , *Salix rehderiana*, *S. ernestii* , *Populus purdomii* seedlings, a few *Astragalus mahoshanicus*, and mosses *Bryum argenteum* , *Grimmia plagiopodia*. The S1, S2, and S3 sites (29, 39 and 49 years, respectively) were dominated, respectively, by small‐, moderate‐, and tall‐sized communities of *H. rhamnoides* , *S. rehderiana*, *S. ernestii* , and *P. purdomii*. The S4 site (61 years) is dominated by *P. purdomii*, *B. utilis*, and *Abies fabri*. The S5 (89 years) and S6 (129 years) sites are dominated, respectively, by middle‐aged and mature *A. fabri* trees. Site S2, with an average tree density of 14.375 (1000 stems ha^−1^), had the highest canopy cover (86%), while S1 and S3 exhibited intermediate and lower cover, respectively (Table 1). Moss communities at the S1, S2, and S3 stages were dominated by *R. japonicum* , *E. pulchellum* , and *C. cirrosum* , respectively (Table 1).

**TABLE 1:** Basic information of the seven successional chronosequences on the glacial retreat area of Hailuogou Glacier.

Site No.	Stand age (yr) until 2019	Latitude	Longitude	Altitude (m a.s.l.)	Distance to glacier (m)	Tree density (1000 stems ha^−1^)	Percent cover of canopy vegetation (%)	Vegetation types	Dominating moss species
S0	4	29°33.915′	101°59.669′	2940	157	86.875	/	Astragalus *mahoshanicus*, Saplings of *Hippophae rhamnoides* , *Salix rehderiana*, *Salix ernestii*, and few *Populus purdomii*	*Bryum argenteum, Grimmia plagiopodia*
S1	29	29°33.947′	101°59.761′	2949	318	25.625	75%	Small trees of *H. rhamnoides* , *S. rehderiana*, *S. ernestii* , and *P. purdomii*	*Racomitrium japonicum*
S2	39	29°33.967′	101°59.813′	2942	411	14.375	86%	Middle sized trees of *H. rhamnoides* , S. rehderiana, *S. ernestii* , and P. purdomii	*Eurhynchium pulchellum*
S3	49	29°34.114′	102°00.036′	2930	858	1.05	58%	Middle sized trees of *H. rhamnoides* , *S. rehderiana*, *S. ernestii* , *P. purdomii*, *Betula utilis* and sparse *H. rhamnoides*	*Cirriphyllum cirrosum*
S4	61	29°34.192′	102°00.185′	2923	1140	0.80	/	Large trees of *S. rehderiana*, *S. ernestii* , *P. purdomii, B. utilis*, and small trees of *Abies fabri*	*Actinothuidium hookeri*
S5	89	29°34.328′	102°00.441′	2883	1620	0.4	/	*Abies fabri* and *Picea brachytyla*	*Pleurozium schreberi*
S6	129	29°34.434′	102°00.640′	2858	2000	0.4	/	*Abies fabri* and *Picea brachytyla*	*Pleurozium schreberi*

### 2.2 Sampling Design

The cover and biomass of moss bottom layer communities were measured at the seven successional sites (S0–S6) in July 2018. At each site, three 10 m × 10 m plots, placed 10 m apart, were established. Within each plot, three 50 cm × 50 cm subplots were positioned along the diagonal line. Moss cover within each subplot was measured using a screen of 50 cm × 50 cm with 400 grids, each with an opening size of 2.5 cm × 2.5 cm. The percentage cover of mosses in the subplot was calculated based on the number of grid cells occupied by mosses (Sun et al. 2013). Moss biomass was determined by clipping and collecting green parts of all mosses within the subplot and measuring their dry weight (Sun et al. 2013).

### 2.3 Reciprocal Transplant Experiment

A reciprocal transplant experiment was conducted at three successional sites (S1, S2, and S3), with distinct canopy vegetation and moss community compositions. To minimize human disturbance, fencing had been established around the sites.

At each successional site, five 5 m × 5 m plots, spaced 3 m apart, were randomly established. Within each plot, three square microcosms (measuring 25 cm × 25 cm) of the dominant moss species (similar in cover and composition) were selected. Each microcosm was assigned to one of the three treatments: control (C), left intact in situ; transplant control (TC), excavated to the humus layer and replanted at its original location to account for transplantation effects (Deluca et al. 2007; Jean, Melvin, et al. 2020); and reciprocal transplant, moved to a neighboring successional stage to isolate canopy‐driven impacts. The reciprocal transplantation included transplanting moss microcosms from S1 to S2, S2 to S1, S2 to S3, and S3 to S2. All microcosms were carefully extracted intact and transplanted within 3 h to minimize disturbance.

### 2.4 Canopy Plant Litter Addition Experiment

The litter addition experiment was conducted concurrently with the reciprocal transplant, using the same experimental plots. Within each of the 5 m × 5 m plots, four additional moss microcosms (25 cm × 25 cm, similar in species composition and initial cover) were selected. Each microcosm was assigned to one of the four treatments: (1) control (C), no litter addition; (2) poplar litter, 10 g (dry weight) of *P. purdomii* leaf litter; (3) willow litter, 10 g (dry weight) of *S. rehderiana* leaf litter; and (4) sea buckthorn litter: 10 g (dry weight) of *H. rhamnoides* leaf litter. Litter quantity was standardized to match the mean annual litterfall (160 g m^−2^) at site S2 (the stage with the lowest moss cover and biomass). Tree litter was evenly distributed atop the moss layer without physical disruption to mimic natural accumulation.

### 2.5 In Situ Acetylene Reduction Assay (ARA)

Nitrogen fixation rates of mosses in both the transplantation and litter addition experiments were quantified monthly in 2018–2019 using acetylene reduction assay (ARA) (Schöllhorn and Burris 1967; Deluca et al. 2008). Prior to measurement, moss carpets in each microcosm were evenly divided into four quadrats, with one randomly selected for ARA measurements. For each measurement, twenty 5 cm apical shoot segments were collected, composited, split into two subsamples, and transferred to two 25 mL vials, each containing 200 μL of sterile water and sealed with rubber septa. To eliminate the effect of endogenous ethylene production in plant tissues (Smercina et al. 2019), half the subsamples were exposed to acetylene exposure, while the other half served as no‐acetylene controls. For acetylene treatment, 2.5 mL of headspace air was replaced with 2.5 mL of acetylene using a syringe, ensuring that 10% of the headspace was filled with acetylene gas (Deluca et al. 2008). To minimize potential artifacts from light and incubation temperature, and to simulate natural N_2_ fixation conditions (White et al. 2020), vials were incubated in situ for 24 h under ambient light and temperature. Headspace gases were then collected and transported to the laboratory for total ethylene production analysis. The headspace volume was determined via water displacement. Ethylene (C_2_H_4_) production in the headspace was quantified using a gas chromatograph (Clarus 680; PerkinElmer, USA) equipped with a flame ionization detector and helium as the carrier gas. Conversion rates of acetylene (C_2_H_2_) to ethylene (C_2_H_4_) were calculated based on changes in C_2_H_4_ concentration in the headspace, with no‐acetylene controls used to correct for endogenous ethylene production.

### 2.6 Moss Cover, Biomass and Photosynthesis Rate

Moss cover in the transplantation and litter addition plots was investigated before and after the experiments using the standardized grid‐based screen method (as previously described). Moss biomass was measured using the harvesting method. Moss photosynthetic rates were measured using a portable photosynthesis system (Li6400, LI‐Cor Inc., Lincoln, USA) fitted with a custom‐built transparent chamber. During measurement, CO_2_ concentration was maintained at 400 μmol mol^−1^ (ambient atmospheric level) with a flow rate of 500 μmol s^−1^; temperature was controlled at 15°C ± 1°C. The chamber was carefully sealed to the moss surface using a flexible foam gasket to prevent air leakage and physical compression of the moss layer.

### 2.7 Environmental Parameters

Daylight hours, temperature, and relative humidity were obtained from the Gongga Mountain Ecological Monitoring Station, within 1 km from the study site. Throughfall N deposition was estimated by monthly collecting throughfall at each of the sites. After collecting, throughfall water was filtered through a 0.45 μm membrane filter (Millipore Corp, USA), extracted with 2 M KCl, and quantified using a continuous flow analyzer (AA3, Seal Analytical, Germany).

### 2.8 Data Analysis

To examine the differences in moss cover and biomass across the glacial chronosequence, a one‐way ANOVA with Tukey's post hoc tests was performed, using site age as the independent variable. Similarly, one‐way ANOVA and independent sample *t*‐tests were employed to assess treatment effects (transplantation or litter addition) on moss cover, biomass, photosynthesis rate, and N_2_‐fixation rates. Linear regression was used to evaluate the correlation between nitrogen fixation rate and photosynthesis with moss biomass (cover). Residual diagnostics were performed using the performance (Lüdecke et al. 2021) and DHARMa (Hartig 2022) packages in R. Log‐transformations were applied to response variables when model assumptions of normality or homoscedasticity were not met. Redundancy analysis (RDA) was performed using the “vegan” package (Borcard et al. 2018). In the first model, N_2_‐fixation and photosynthesis rates were used as the response matrix, with daylight hours, temperature, and TF–${\mathrm{NH}}_{4}^{+}$ concentration as the predictor matrix, to assess the effects of environmental factors on seasonal variations in moss N_2_‐fixation and photosynthetic activity (monthly scale). In the second model, moss biomass and cover were used as the response matrix, with moss N_2_‐fixation rate, photosynthesis rate, litter biomass, and throughfall ${\mathrm{NH}}_{4}^{+}$ concentration as the predictor matrix, to explore how moss cover and biomass respond to functional processes and environmental drivers. Multicollinearity among predictor variables was evaluated using the “car” package in R (Fox and Weisberg 2019), and highly collinear variables were excluded from the final RDA models (Montgomery and Peck 1992).

## 3 Results

### 3.1 Influence of Successional Stage on Moss Bottom Layer Dynamics

**FIGURE 2:**
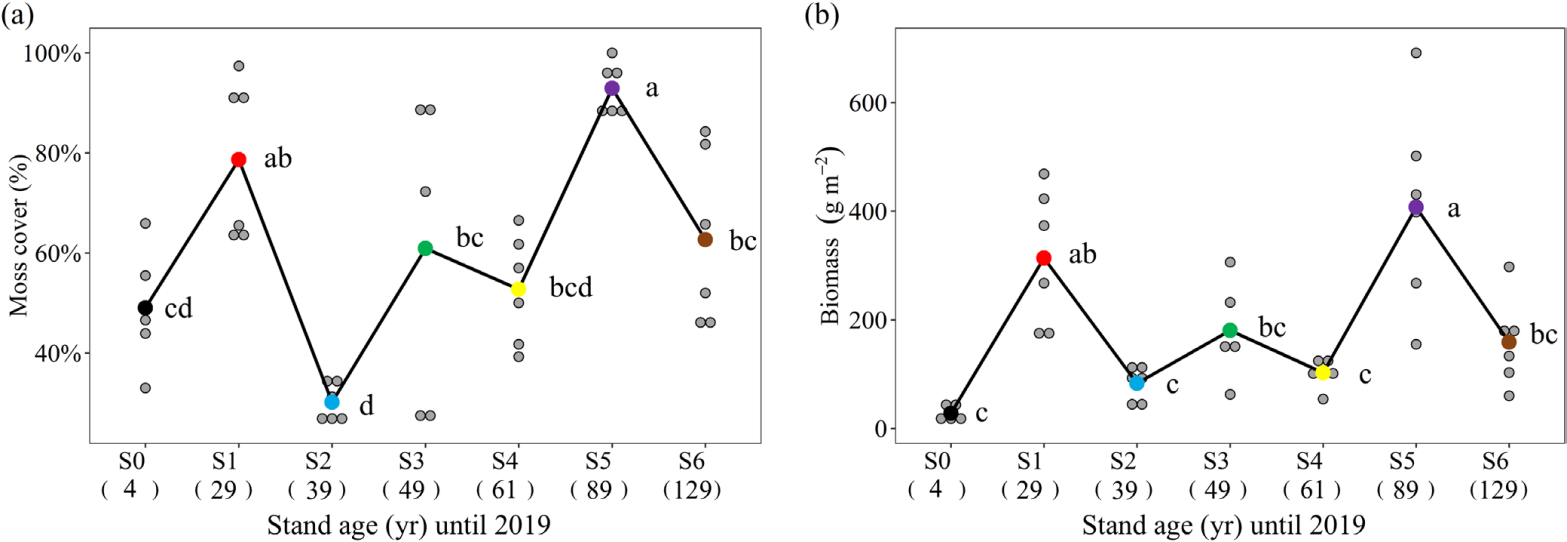
Dynamics of moss cover (a) and moss biomass (b) along the successional chronosequence (S0–S6) on the glacial retreat area of Hailuogou glacier. Gray dots represent sampling data, and middle dots of different colors represent the means (*n* = 5). Different lowercase letters indicate significant differences between successional stages (*p* < 0.05).

Moss layer cover and biomass varied substantially across successional stages (*F* (6, 33) = 10.07, *p* < 0.001; *F* (6, 33) = 10.43, *p* < 0.001). Both moss cover and biomass were low at the initial stage (S0), gradually increased with succession, and peaked at S1 (cover: 78.7% ± 6.5%; biomass: 313.9 ± 51.7 g m^−2^). As succession progressed, moss cover and biomass declined sharply, reaching their lowest values at S2 (cover: 30.2% ± 1.5%; biomass: 82.9 ± 13.1 g m^−2^). By S5, they peaked again at 92.9% ± 2.1% and 407.6 ± 76.1 g m^−2^, respectively. Overall, moss cover and biomass followed a multi‐peak pattern, with high biomass associated with high cover, indicating parallel trends (Figure 2).

### 3.2 Canopy Effects on the Moss Bottom Layer in a Reciprocal Transplant Experiment

**FIGURE 3:**
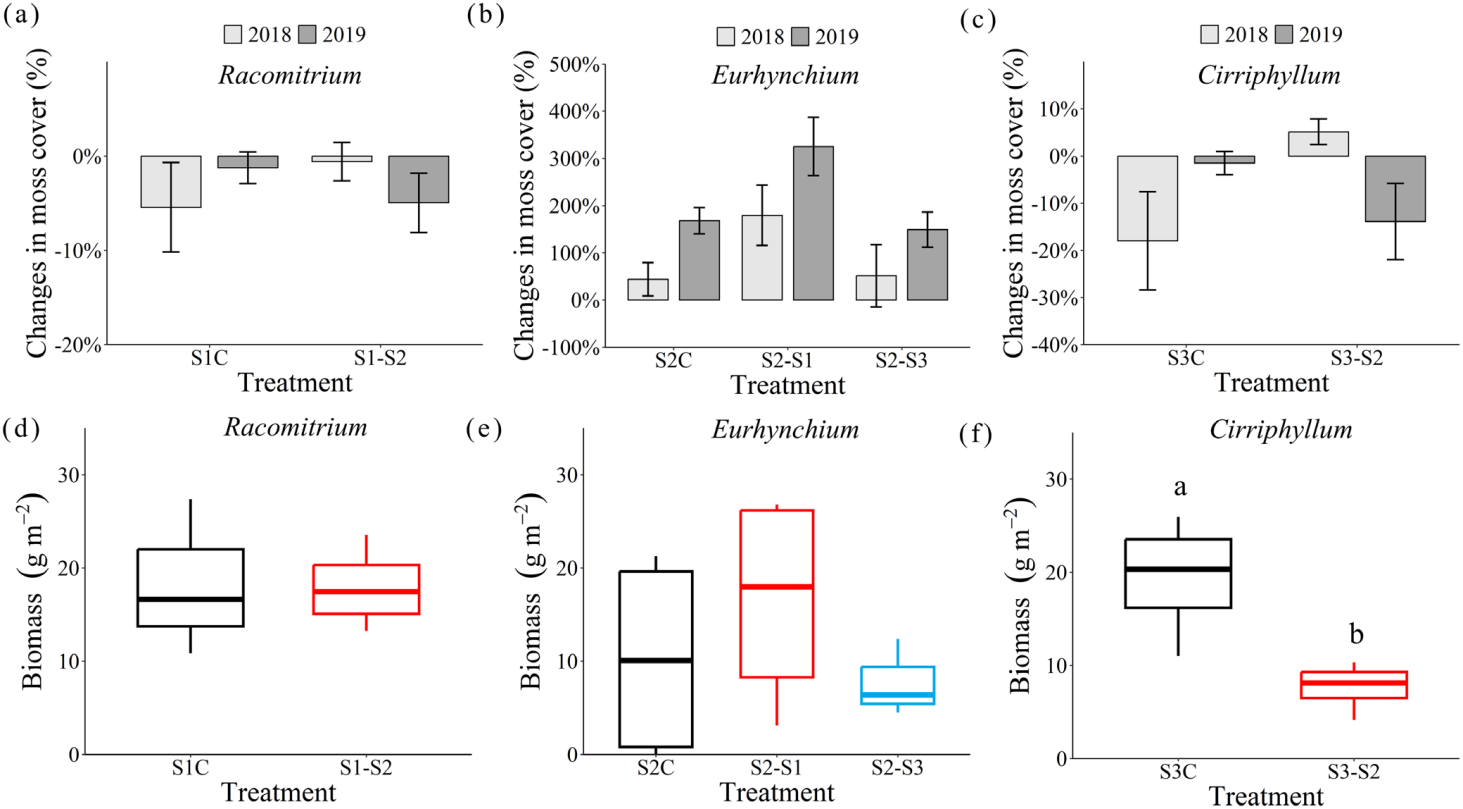
Changes in moss cover (a–c) and biomass (d–f) after reciprocal moss transplantation. S1C, S2C, and S3C represent intact control moss microcosms at the S1 (*Racomitrium*), S2 (*Eurhynchium*), and S3 sites (*Cirriphyllum*), respectively (mean ± SE, *n* = 5). S1–S2, transplanting moss microcosms from site 1 (S1) to site 2 (S2); S2–S1, from site 2 to site 1; S2–S3, from site 2 to site 3; S3–S2, from site 3 to site 2. Different lowercase letters indicate significant differences between transplantation treatments (*p* < 0.05).

Reciprocal transplantation of moss microcosms altered their cover and biomass. Transplantation of *Racomitrium* moss carpets from S1 (29‐year stage) into S2 (39‐year stage with dense canopy) (Figure 3a,d) and *Cirriphyllum* from S3 (49‐year stage with open canopy) into S2 (Figure 3c,f) reduced moss cover and biomass. Specifically, *Cirriphyllum* had a 60% decline in cover and a 14% reduction in biomass (*t* (6) = 3.35, *p* = 0.015), with these changes being more pronounced than those observed for *Racomitrium*. In contrast, *Eurhynchium* moss transplanted from S2 into S1 and S3 increased through time. When introduced into S1, *Eurhynchium* cover and biomass increased by 325% and 59%, respectively, while transplantation into S3 increased its cover by 49% and biomass by 60% (Figure 3b,e).

**FIGURE 4:**
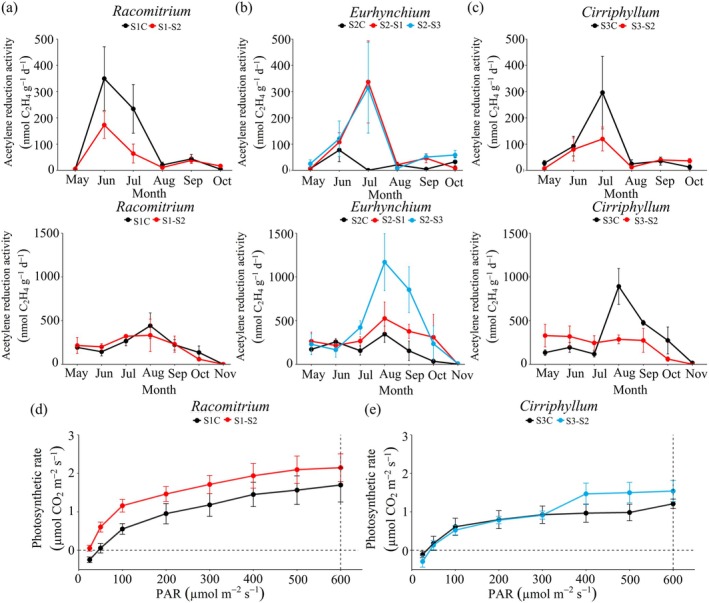
Effects of transplantation on dominant moss N_2_‐fixation rates (as estimated by acetylene reduction) (mean ± SE, *n* = 5) in (a) *Racomitrium*, (b) *Eurhynchium*, and (c) *Cirriphyllum*. The vertical graphs represent measurements from 2018 (top) and 2019 (bottom). Effect of transplantation on moss photosynthesis rates (mean ± SE, *n* = 5) in (d) *Racomitrium* and (e) *Cirriphyllum*. Different lowercase letters indicate significant differences between treatments (*p* < 0.05).

N_2_‐fixation rates varied among moss species, forest types, and transplantation treatments (Figure 4a–c). In native control plots, *Cirriphyllum* exhibited the highest N_2_‐fixation rates (199.27 ± 69.26 C_2_H_4_ nmol g^−1^ day^−1^), followed by *Racomitrium* (155.27 ± 37.88 C_2_H_4_ nmol g^−1^ day^−1^) and *Eurhynchium* (97.36 ± 30.97 C_2_H_4_ nmol g^−1^ day^−1^). Transplantation of *Racomitrium* (from S1) and *Cirriphyllum* (from S3) into S2 reduced N_2_‐fixation rates by 17.2% and 30.2%, respectively (Figure 4a,c). Conversely, *Eurhynchium* transplanted from S2 into S1 and S3 showed increased N_2_‐fixation rates (96.5% and 189.4%, respectively) (Figure 4b).

*Racomitrium* transplanted from S1 into S2 had a 55% increase in photosynthesis rates. However, for *Cirriphyllum* transplanted from S3 into S2, photosynthetic rates only increased when light intensity exceeded 400 μmol m^−2^ s^−1^ (Figure 4d,e).

### 3.3 Effects of Canopy Litter Addition on Moss Bottom Layer

**FIGURE 5:**
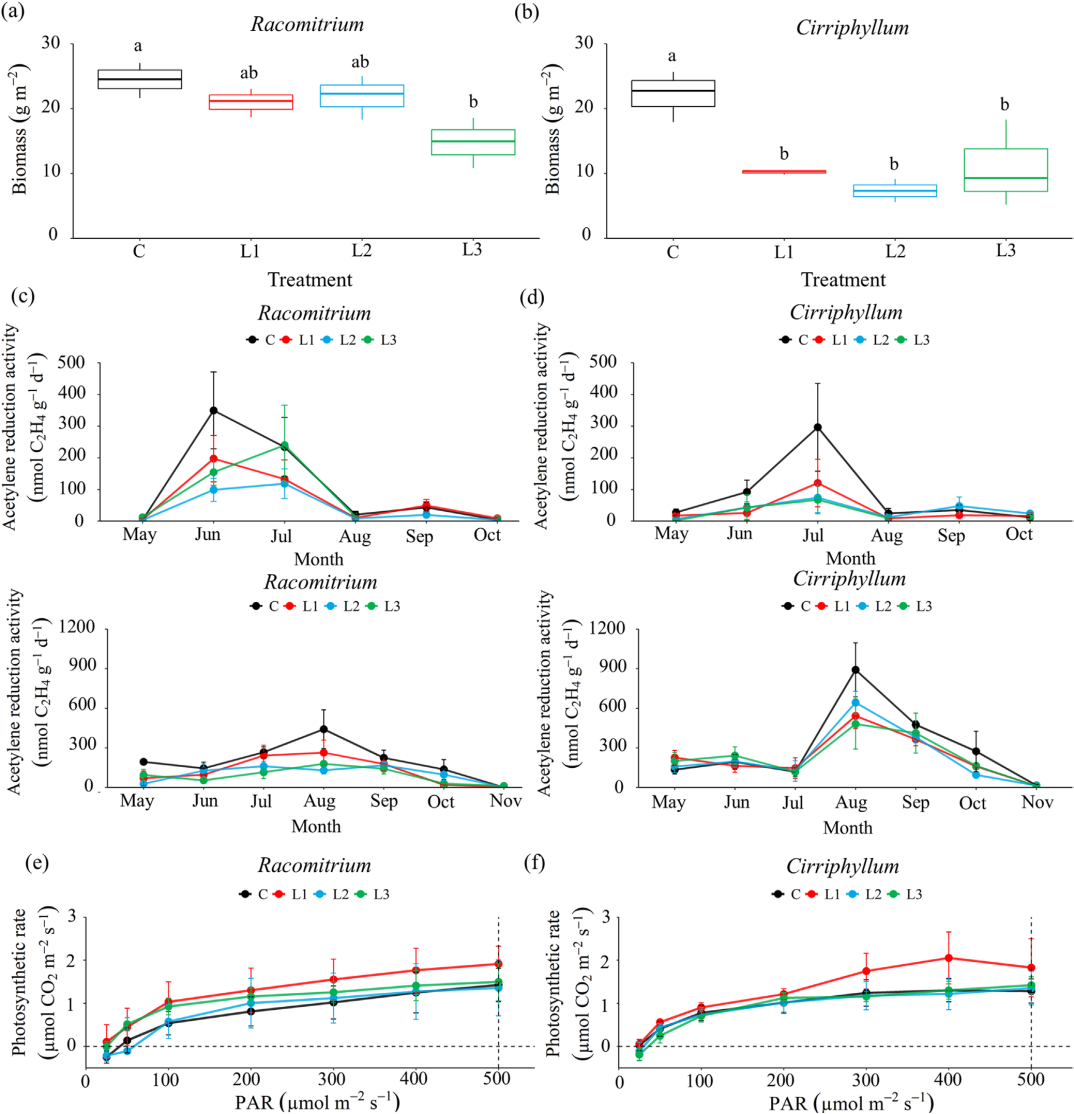
Effects of canopy tree litter addition on moss biomass (a, b), N_2_‐fixation rates (as estimated by acetylene reduction) (c, d), and photosynthesis rates (e, f) (mean ± SE, *n* = 5). Panels (c, d): Vertical graphs represent measurements from 2018 (top) and 2019 (bottom). L1, Poplar leaf litter; L2, Willow leaf litter; L3, Sea buckthorn leaf litter. Different lowercase letters indicate significant differences between treatments (*p* < 0.05).

Canopy leaf litter addition decreased moss N_2_‐fixation rates, with willow litter having the strongest inhibitory effect. *Racomitrium* was more sensitive to litter addition than *Cirriphyllum*, exhibiting a 38.0%–53.1% reduction in N_2_‐fixation rates (Figure 5c), compared to a 26.2%–29.7% decrease in *Cirriphyllum* (Figure 5d). Litter addition also negatively impacted moss biomass, though the magnitude of the effect varied by litter type and species (*Racomitrium*: *F* (3, 8) = 33.77, *p* < 0.001, *η* ^2^ = 0.93; *Cirriphyllum*: (*F* (3, 8) = 20.44, *p* < 0.001, *η* ^2^ = 0.88)). Sea buckthorn leaf litter caused the greatest biomass reduction in *Racomitrium* (46.3% decrease), whereas willow leaf litter had the strongest negative effect on *Cirriphyllum* (70.0% decrease) (Figure 5a,b). In contrast, litter addition did not suppress photosynthetic rates. Instead, poplar leaf litter enhanced photosynthetic activity by 54.7% in *Racomitrium* and 38.4% in *Cirriphyllum* (Figure 5e,f).

### 3.4 Direct and Indirect Factors Controlling Moss Growth

**FIGURE 6:**
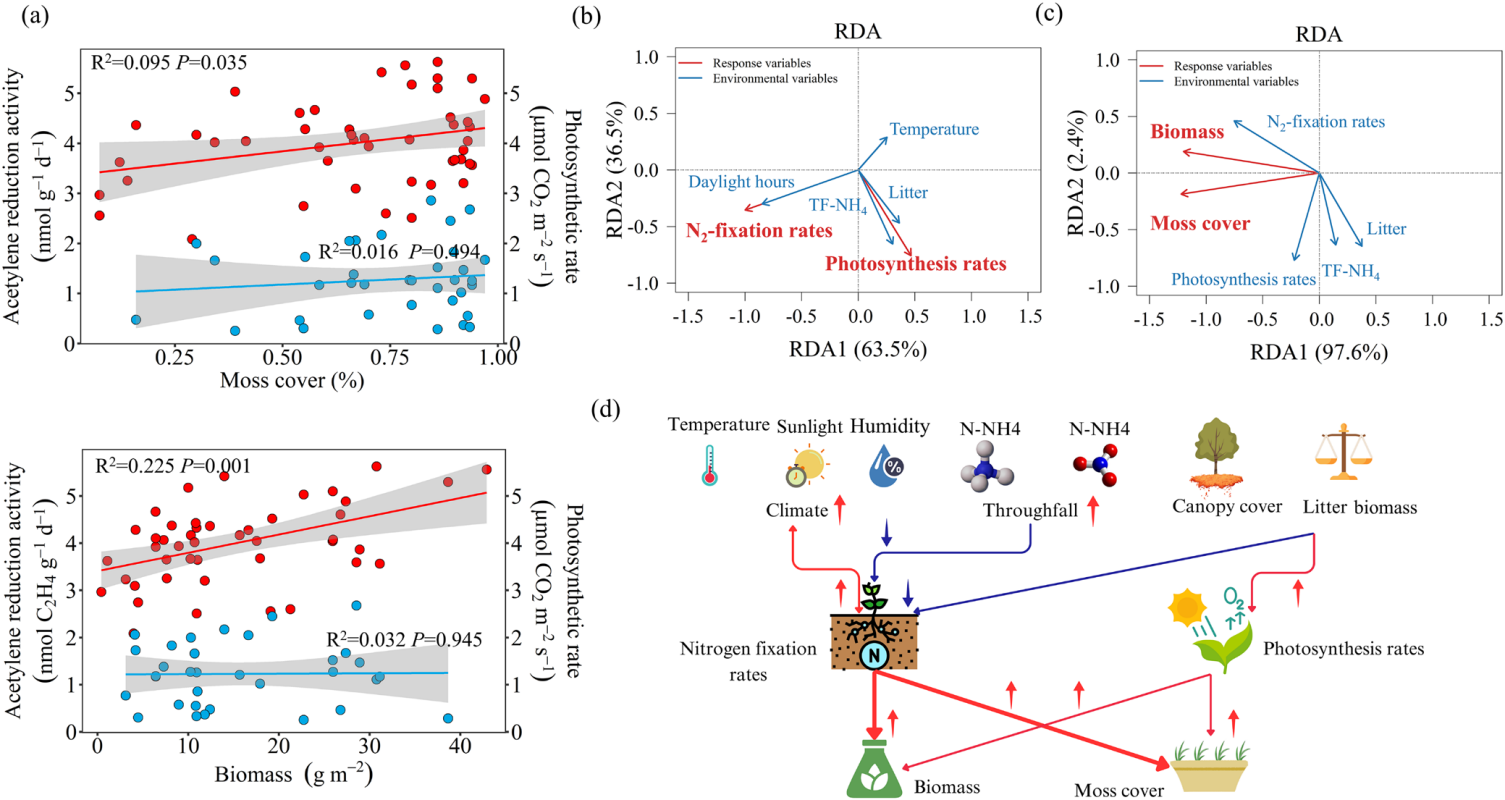
Linear regression analysis of moss biomass and coverage (a) with the mean acetylene reduction value and photosynthetic rate across successional stages on the glacial retreat area of Hailuogou Glacier (Red dots represent the mean acetylene reduction value, blue dots represent the photosynthetic rate, and shaded areas indicate the 95% confidence interval); (b) Redundancy Analysis (RDA) showing the effects and relative contributions of daylight hours, temperature, and throughfall ammonium (TF–${\mathrm{NH}}_{4}^{+}$) on moss N_2_‐fixation and photosynthetic rates; (c) RDA showing moss cover and biomass responses to N_2_‐fixation, photosynthetic rates, litter biomass, and TF–${\mathrm{NH}}_{4}^{+}$; (d) Conceptual model illustrating the integrated mechanisms through which climate, throughfall, and canopy litter impact moss growth by affecting N_2_‐fixation and photosynthetic rates.

During the primary successional chronosequence, moss cover and biomass were positively correlated with N_2_‐fixation rates as estimated by the ARA method. Moss cover showed a significant positive correlation with N_2_‐fixation (*F* (1, 45) = 4.71, *p* = 0.035, *R* ^2^ = 0.095; slope = 0.096 ± 0.044), while biomass had a stronger relationship (*F* (1, 43) = 12.47, *p* = 0.001, *R* ^2^ = 0.225; slope = 5.80 ± 1.64). In contrast, no significant relationship was observed with moss photosynthesis rates. Variation in moss N_2_‐fixation rates explained 22.7% of the variation in biomass and 9.5% of the variation in moss cover (Figure 6a). Given the negligible influence of soil nutrient availability on moss growth, these factors were excluded from further analysis (Figure S4). RDA results revealed that moss biomass and cover were primarily driven by N_2_‐fixation, litter biomass, and throughfall nitrogen input (Figure 6c). Environmental variables collectively explained 20.9% of the variance in moss N_2_‐fixation and photosynthetic rates (*F* (4, 38) = 2.50, *p* = 0.038). Among these, daylight hours were identified as the strongest predictor (*F* (1, 38) = 4.85, *p* = 0.013), followed by throughfall ${\mathrm{NH}}_{4}^{+}$ (*F* (1, 38) = 2.78, *p* = 0.068) (Figure 6b). Subsequent analysis revealed that N_2_‐fixation correlated positively with relative humidity but inversely with daylight hours and throughfall N levels (Figure S4). Conversely, photosynthetic rates showed strong positive correlations with throughfall ${\mathrm{NH}}_{4}^{+}$–N concentrations (Figure S5).

## 4 Discussion

### 4.1 Influence of Successional Stage on Moss Bottom Layer Dynamics

We found that moss cover and biomass did not increase progressively, as predicted by the traditional relay floristic model. Instead, they fluctuated over time since glacial retreat, contradicting our first hypothesis and contrasting with observations from other glacier forelands (Irene et al. 2011; Matthews and Vater 2015).

In the very early stages of glacial retreat (S0), harsh abiotic conditions, including nutrient scarcity, extreme temperature fluctuations, high UV exposure, and desiccation stress, limited colonization to a few highly stress‐tolerant species with substantial phenotypic plasticity (Turetsky et al. 2012). Consequently, moss diversity, cover, and biomass remained low at this stage. As succession progressed, vascular plant establishment improved soil conditions, facilitating increased moss cover (Frenot et al. 1998). Moderated light availability and reduced evaporative water loss of the moss layer due to colonization by vascular plants may have further supported the expansion of additional moss species (Turetsky et al. 2012; Kidron and Benenson 2014). However, physical factors (e.g., shading and barrier to growth) and chemical effects (e.g., allelopathy) of broadleaf litterfall (Natalia et al. 2008; Rousk and Michelsen 2017; Jean, Holland‐Moritz, et al. 2020) likely contributed to the sharp decline in moss cover and biomass at S2 by limiting light availability, water retention, and nutrient access (Lange and Green 2005; Li et al. 2017; Jaszczuk et al. 2023). In later stages, forest structure changes such as reduced tree density due to self‐thinning and the formation of canopy gaps may allow partial recovery of forest‐floor mosses through reduced litter accumulation and improved light availability.

### 4.2 Canopy Effects on Moss Bottom Layer in a Reciprocal Transplant Experiment

The N_2_‐fixation rate, photosynthetic rate, and growth of the moss bottom layer were sensitive to canopy and seasonal shifts, consistent with findings by Bjerke et al. (2013), Rousk and Michelsen (2017) and Permin et al. (2021). Compared to the closed canopy of S2, the relatively open canopies and presence of forest gaps in S1 and S3 may maintain higher relative humidity while allowing for greater sunlight penetration (Belnap 2003; Stewart et al. 2014), creating favorable conditions for moss N_2_‐fixation, photosynthesis, and growth (Serpe et al. 2013; Calabria et al. 2020; Jean, Holland‐Moritz, et al. 2020). Seasonal fluctuations in N_2_‐fixation rates appeared to coincide with variation in temperature and humidity in the corresponding years (Figure S1).

Reciprocal transplantation revealed that mosses transplanted from early‐ (S1, *Racomitrium*) and mid‐succession (S3, *Cirriphyllum*) into closed‐canopy stage (S2) had increased photosynthetic rates, but sharply reduced N_2_‐fixation rates, cover, and biomass, likely due to the elevated N deposition under the dense canopy. In contrast, mosses transplanted from S2 (*Eurhynchium*) into S1 and S3 had higher N_2_‐fixation rates, cover, and biomass, as shown by Deluca et al. (2007). This result suggests that moss N_2_‐fixation and growth are strongly influenced by successional‐stage‐dependent environmental factors.

Mechanisms underlying canopy vegetation effects and interspecific differences in moss N_2_‐fixation across successional stages remain poorly understood. Unlike secondary succession studies (Deluca et al. 2007), where mosses transplanted from early to late stages exhibited a gradually increasing N_2_‐fixation rate, our primary succession study showed higher N_2_‐fixation rates when mosses were transplanted to S1 and S3. This discrepancy likely stems from contrasting trends in N availability across the successional chronosequence. In Deluca et al. (2007), post‐fire secondary successional forests had high available N and dense canopy in early stages, whereas in our system, canopy density and throughfall N were highest at S2. These results indicate that moss N_2_‐fixation rates do not follow a universal successional trend but depend on canopy vegetation properties.

For instance, reduced light availability under denser canopy may limit moss N_2_‐fixation efficiency (Kox et al. 2020). Elevated throughfall N from dense canopies may suppress the colonization or activity of moss‐associated cyanobacteria (Deluca et al. 2007, 2008; Sorensen et al. 2012; Salemaa et al. 2019), thereby reducing N_2_‐fixation rates. Furthermore, interspecific differences in N_2_‐fixation rates among mosses at the same site (e.g., *Racomitrium* natively at S1 vs. *Eurhynchium* transplanted from S2 to S1; *Eurhynchium* natively at S2 vs. *Racomitrium* transplanted from S1 to S2 and *Cirriphyllum* from S3 into S2; *Cirriphyllum* natively at S3 vs. *Eurhynchium* transplanted from S2 into S3) demonstrate that moss species are a key determinant of N_2_‐fixation rates (Figure S3). This variation may arise from species‐specific control over cyanobacterial colonization (Bay et al. 2013) or cyanobacteria metabolic shifts toward utilizing available N rather than fixing N_2_ under high N availability (Meeks 1998).

In contrast to N_2_ fixation, photosynthesis rates increased when mosses were transplanted from S1 or S3 into S2. This likely happened due to the denser canopy at S2, mitigating heat stress, reducing photoinhibition, and maintaining higher long‐term moisture levels (Sonesson et al. 1992; Murray et al. 1993; Li et al. 2017). Furthermore, elevated N content in throughfall as well as potentially greater nutrient leaching at S2 (Figure S2) relative to S1 and S3 may enhance nutrient availability, further supporting moss photosynthesis (Van den Elzen et al. 2020).

### 4.3 Effect of Canopy Litter Addition on Moss Bottom Layer

In contrast to Serpe et al. (2013), our results indicate that canopy plant litter addition moderately promoted photosynthesis in the moss bottom layer, which aligns with the photosynthetic responses observed in transplantation experiments. This stimulatory effect is possibly related to the microclimate modulation, which might include providing shading, reducing summer heat stress, and photoinhibition (Sonesson et al. 1992; Murray et al. 1993; Li et al. 2017). Furthermore, leached nitrogen and phosphorus from litter may supplement essential nutrients for moss photosynthesis (Rousk and Michelsen 2017; Van den Elzen et al. 2020). The strongest positive effect of *Poplar* litter was likely related to its higher P concentration and balanced C:N:P ratios compared to other species.

Despite enhanced photosynthesis, litter addition did not increase moss cover and biomass. In contrast, biomass declined, aligning with boreal forest studies using birch and aspen litter (Natalia et al. 2008; Jean, Holland‐Moritz, et al. 2020). This result supports the hypothesis that deciduous broadleaf litter negatively impacts moss viability. Further, N_2_‐fixation rates were also suppressed under canopy litter addition, consistent with observations for *H. splendens* and *Pleurozium schreberi* (Jean, Melvin, et al. 2020). These might be related to the N‐mediated suppression of plant litters, that is, elevated N availability from litter leachates reduces moss reliance on N_2_‐fixation (Deluca et al. 2007, 2008). Additionally, litter decomposition rates and polyphenolic and tannin compounds in litter may impair cyanobacteria colonization or activity (Cornwell et al. 2008; Natalia et al. 2008; Rousk and Michelsen 2017; Jean, Holland‐Moritz, et al. 2020), thereby depressing N_2_‐fixation. The stronger negative effects of willow litter relative to other species were in accordance with its higher phenol and tannin content (Table S1), suggesting species‐specific inhibition of cyanobacteria symbionts and N_2_‐fixation. These findings parallel subarctic tundra studies, in which birch litter enhanced, while willow litter suppressed moss N_2_‐fixation (Rousk and Michelsen 2017). The dissociation between photosynthetic stimulation and biomass decline suggested other mechanisms, such as barrier effects (Jean, Melvin, et al. 2020) as potential pathways in affecting moss growth and colonization.

### 4.4 Mechanisms of Canopy Influence on Moss Bottom Layer Growth

We found that changes in moss N_2_‐fixation and photosynthesis rates were correlated with daylength regimes, thereby extending empirical support from Calabria et al. (2020) to subalpine ecosystems. Beyond photoperiodicity, throughfall ${\mathrm{NH}}_{4}^{+}$–N and ${\mathrm{NO}}_{3}^{-}$–N concentrations, as well as relative humidity, emerged as key environmental modulators of moss N_2_‐fixation and photosynthesis rates. These findings align with previous studies conducted in northern European and North American boreal forests (Gundale et al. 2012, 2013; Whiteley and Gonzalez 2016; Salemaa et al. 2019). Notably, soil nutrient status showed minimal correlation with moss biomass or cover. This is likely because terrestrial mosses primarily obtain water and nutrients from atmospheric deposition (precipitation and dry deposition), with minimal uptake from substrates due to their nonvascular nature.

Although moss biomass variation correlated with N_2_‐fixation rates, photosynthetic activity remained decoupled from productivity metrics. The divergence is potentially attributed to canopy litter's physical barrier effects, which restrict moss colonization and biomass accrual despite photosynthetic upregulation. This critical dissociation between carbon assimilation and N economy underscores how canopy‐structured abiotic filters (e.g., light attenuation, hydrological modification, and throughfall N deposition) exert asymmetric control over N_2_‐fixation relative to photosynthesis. Multivariate analysis identified litter biomass and throughfall N as the paramount regulators of moss cover and biomass (Figure 6c). Successional progression toward denser canopies amplified litter production and throughfall N concentrations, enhancing understory shading, humidity retention, and nutrient availability. The resultant decoupling of moss photosynthetic potential from realized productivity reveals a potentially novel successional paradox, that is, canopy closure enhances moss carbon assimilation capacity while simultaneously constraining biomass accumulation through N economy and colonization barriers.

## 5 Conclusion

This study elucidates the complex growth dynamics and functional mechanisms governing moss bottom layer development across a primary successional chronosequence, providing insights into ecosystem reorganization under climate‐driven acceleration of alpine glacial retreat. Our findings demonstrate that successional shifts in canopy composition and density drive changes in abiotic factors, such as light availability, throughfall N, and canopy litter accumulation, which in turn regulate N_2_‐fixation rates, photosynthetic activity, and moss bottom layer cover and biomass trajectories following glacial retreat. Our results provide compelling evidence for a strong coupling between moss biomass accumulation and N_2_‐fixation, as well as a decoupling of moss photosynthesis from overall moss productivity along the glacial primary succession in subalpine forests. Successional shifts in canopy composition and cover directly influence moss productivity, photosynthesis, and N_2_‐fixation rates, highlighting a dynamic interplay between canopy and moss bottom layer interactions. These interactions may play a pivotal role in shaping the patterns and functions of subalpine forest development.

## Author Contributions

**Jie Deng:** investigation (equal), validation (equal), visualization (equal), writing – original draft (equal), writing – review and editing (equal). **Genxu Wang:** conceptualization (equal), methodology (equal), resources (equal). **Shouqin Sun:** funding acquisition (equal), methodology (equal), supervision (equal), validation (equal), writing – review and editing (equal). **Wentian Xie:** investigation (equal), resources (equal), software (equal). **Feng Long:** investigation (equal), project administration (equal), software (equal). **Zhaoyong Hu:** supervision (equal), validation (equal). **Juying Sun:** supervision (equal), validation (equal). **Xiangyang Sun:** supervision (equal), validation (equal). **Thomas H. DeLuca:** software (equal), supervision (equal), writing – review and editing (equal).

## Conflicts of Interest

The authors declare no conflicts of interest.

## Supporting information


Data S1.

## Data Availability

All data have been archived and are publicly available on the Dryad data repository: https://doi.org/10.5061/dryad.573n5tbkf.
