# Supplementary material for: Canopy‐Mediated Dynamics of Moss Communities in Primary Succession: Coupling of N2 ‐Fixation and Biomass Accumulation in Subalpine Forests Following Glacial Retreat
Source: Ecol Evol. 2025 Jul 11;15(7):e71763. doi: 10.1002/ece3.71763 (PMC12246726; doi:10.1002/ece3.71763)
Supplement: Supplementary file 1 — Data S1. [file ECE3-15-e71763-s001.docx]

**
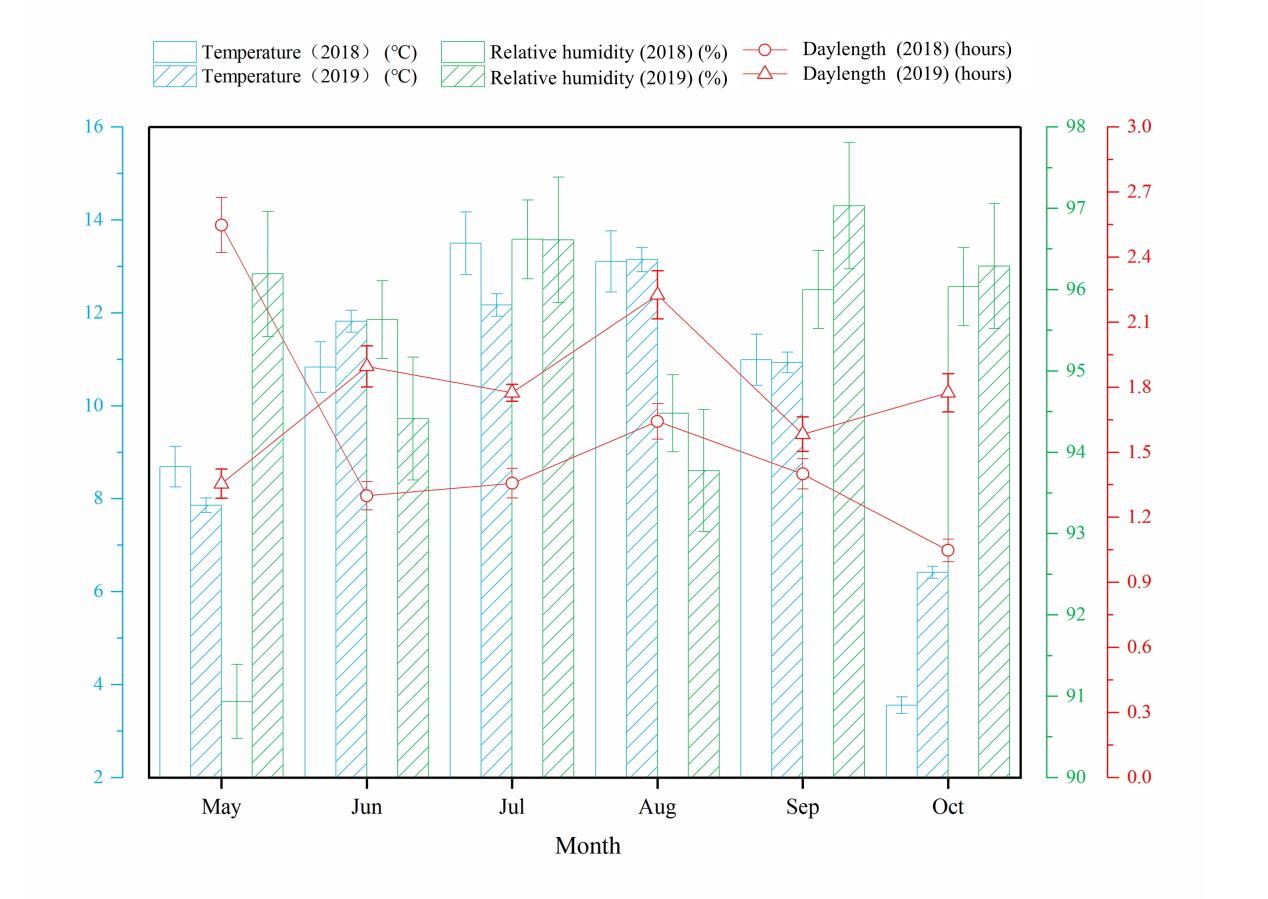
Fig. S1** Gongga Mountain Climate Observatory data (Temperature, Relative humidity

and Daylength changes) for 2018 and 2019


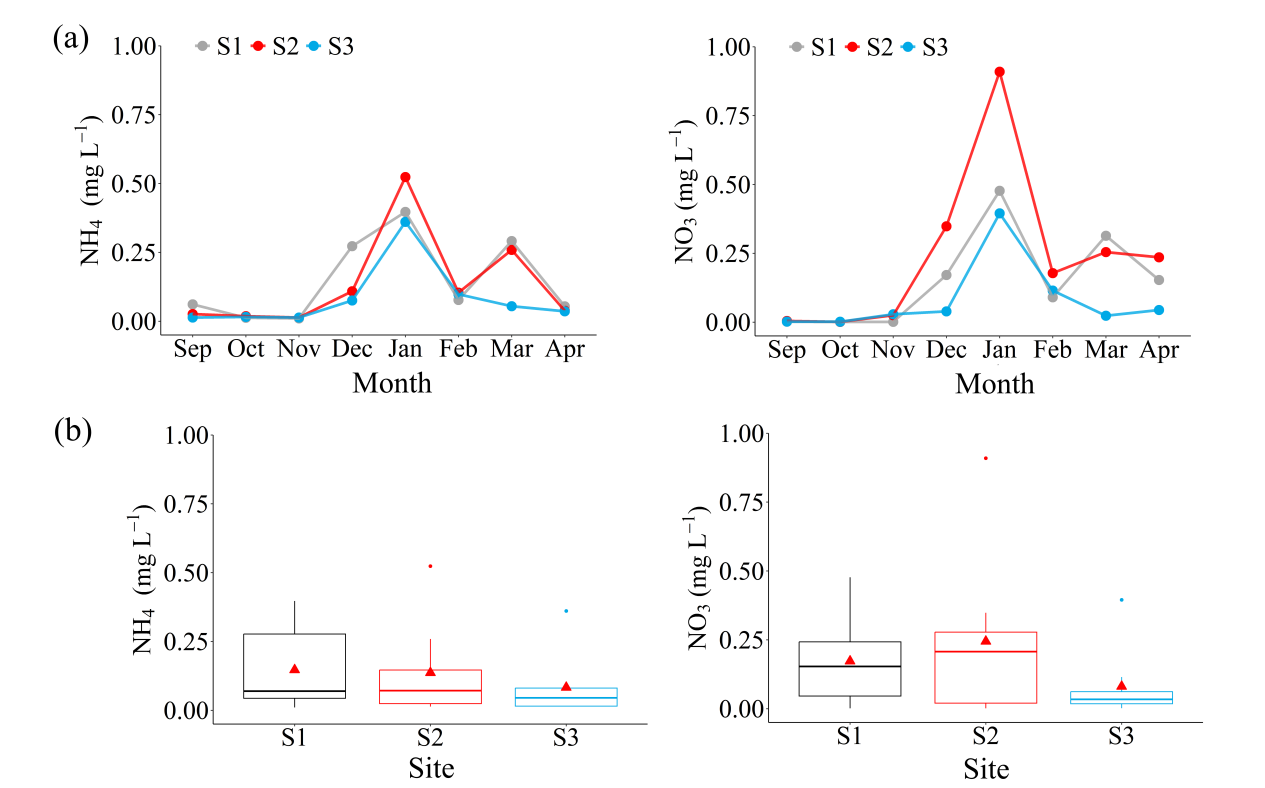


**Fig. S2** Throughfall N analysis of three key successional stages in the glacial retreat zone (c) monthly seasonal changes (d) overall throughfall N input. Different lowercase letters indicate significant differences between treatments (P < 0.05). Data are mean ± SE, n=5.


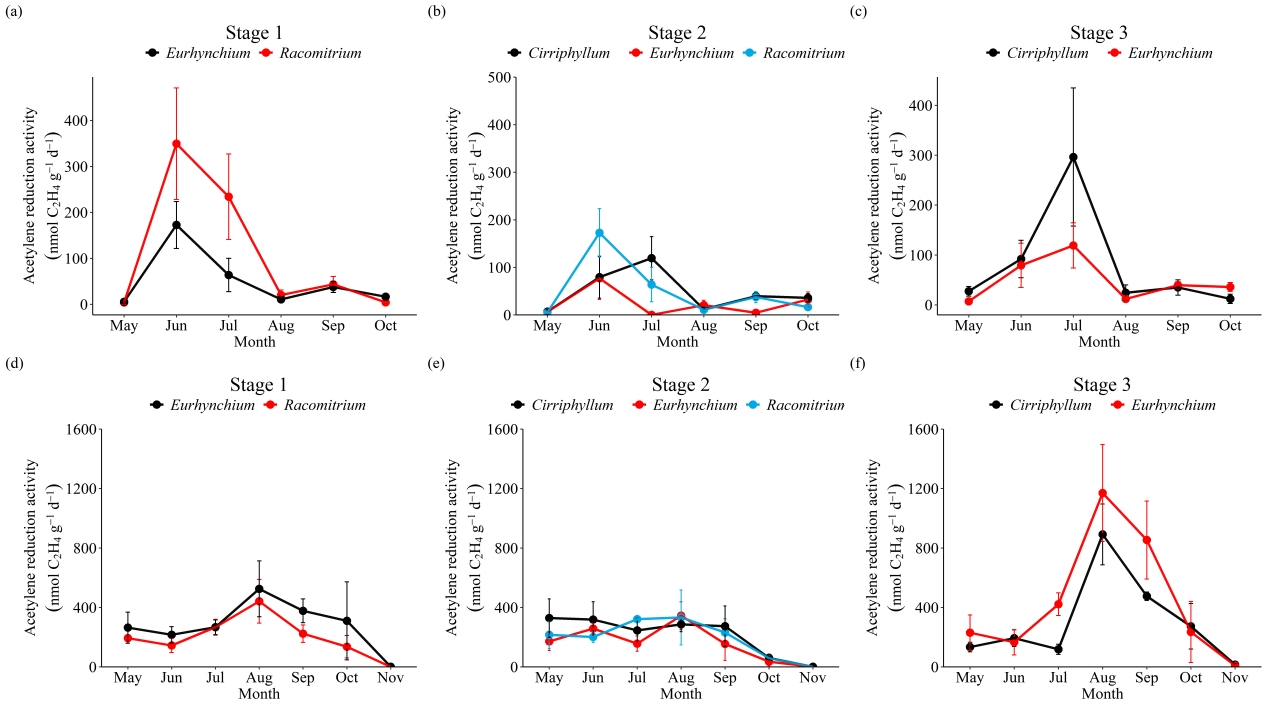


**Fig. S3** Comparison of nitrogen fixation rates (estimated from acetylene reduction) of different species of mosses transplanted to the same chronosequence in 2018 (a-c) and 2019 (d-f) (mean ± SE, n=5). (a) and (d), Stage 1 (S1); (b) and (e), Stage 2 (S2); (c) and (f), Stage 3 (S3).

**
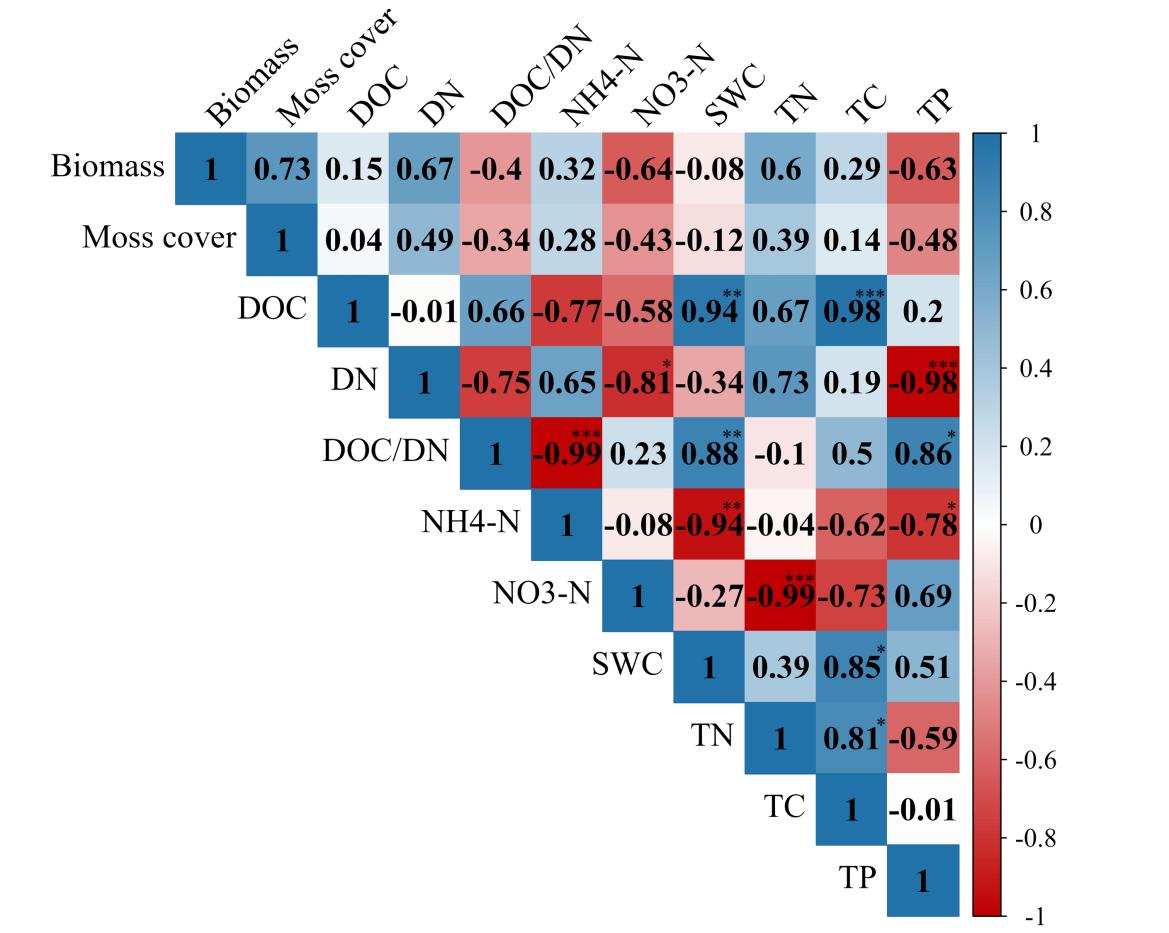
**

**Fig. S4** Moss Growth and Soil Properties: Pearson Correlation Analysis with Two-Tailed Significance Test.


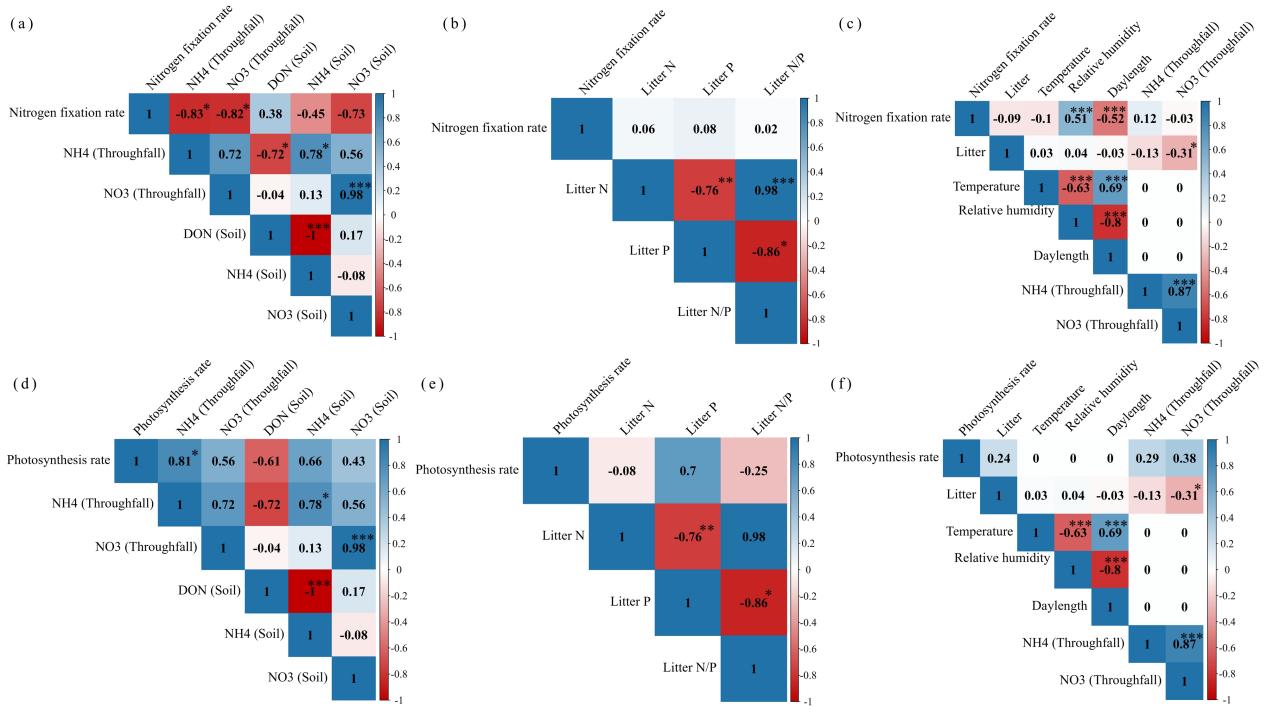


**Fig. S5** Correlation heatmap (Pearson's correlation coefficients) of moss nitrogen fixation rates(a-c) and photosynthesis rates(d-f) with litter nitrogen and phosphorus content, throughfall nitrogen input, soil nitrogen content and environmental variables.

**Table S1.** Chemical properties of canopy plant leaf litters collected from plots on the glacial retreat area of Hailuogou Glacier.

| Litter | Total Phenol  (mg g^-1^) | Tannin  (mg g^-1^) | N  (mg g^-1^) | C  (mg g^-1^) | P  (mg g^-1^) | C/N | C/P | N/P |
| --- | --- | --- | --- | --- | --- | --- | --- | --- |
| Sea buckthorn | 55.50 | 13.98 | 23.64 | 448.96 | 9.19 | 19.01 | 48.85 | 25.72 |
| Willow | 152.82 | 51.17 | 13.30 | 465.23 | 10.54 | 35.05 | 44.20 | 12.64 |
| Poplar | 53.12 | 15.20 | 15.99 | 477.61 | 11.61 | 29.90 | 41.17 | 13.78 |
